# Effects of sorghum residue in presence of pre-emergence herbicides on emergence and biomass of *Echinochloa colona* and *Chloris virgata*

**DOI:** 10.1371/journal.pone.0229817

**Published:** 2020-03-02

**Authors:** Ahmadreza Mobli, Abhimanyu Rinwa, Sahil, Bhagirath Singh Chauhan

**Affiliations:** 1 Department of Agrotechnology, Faculty of Agriculture, Ferdowsi University of Mashhad, Mashhad, Iran; 2 The Centre for Crop Science, Queensland Alliance for Agriculture and Food Innovation (QAAFI), The University of Queensland, Gatton, Queensland, Australia; 3 Department of Agronomy, Punjab Agricultural University, Ludhiana, Punjab, India; 4 Mata Gujri College, Punjabi University of Patiala, Fatehgarh Sahib, Punjab, Punjab, India; Ohio State University South Centers, UNITED STATES

## Abstract

In conservation agriculture systems, farmers gain many advantages from retaining crop residue on the soil surface, but crop residue retention in these systems may intervene with the activity of pre-emergence herbicides. A pot study was conducted to evaluate the effect of different rates of pre-emergence herbicides [imazethapyr (100 and 150 g a. i. ha^-1^), isoxaflutole (100 and 200 g a. i. ha^-1^), metolachlor (1.5 and 2.25 kg a. i. ha^-1^), pendimethalin (2.25 and 3.38 kg a. i. ha^-1^) and prosulfocarb + metolachlor (2.5 and 3.75 kg a. i. ha^-1^)] on seedling emergence and biomass of *Echinochloa colona* and *Chloris virgata* when applied in the presence of sorghum residue at rates equivalent to (0, 3 and 6 t ha^-1^). When seeds of *E*. *colona and C*. *virgata* were not covered with sorghum residue, the seedling emergence and biomass of both weeds was inhibited by 93–100% and 56–100%, respectively, with the application (both rates) of isoxaflutole, metolachlor, pendimethalin and prosulfocarb + metolachlor. Using sorghum residue resulted in lower herbicide efficacy on both weeds. At 3 t ha^-1^ sorghum residue, *E*. *colona* emergence and biomass reduced by 38–100% and 30–100%, respectively, with application of isoxaflutole, metolachlor and pendimethalin (both rates) in comparison with the no-herbicide treatment. Similarly, the emergence and biomass of *C*. *virgata* was also reduced by 92–100% and 25–100%, respectively. The results of this study suggest that crop residue may influence efficacy of commonly used pre-emergence herbicides and that the amount of crop residue on the soil surface should be adjusted according to the nature of the pre-emergence herbicides to achieve adequate weed control.

## Introduction

*Echinochloa colona* (L.) Link and *Chloris virgata* Sw. are major C_4_ summer grass weeds) in Australia, where infested 111000 and 118000 hectares of Australian farms, respectively, causing respective revenue losses of AUD 14.7 and 7.7 million per year [1]. *E*. *colona* is a mimic weed of rice (*Oryza sativa* L.) and is difficult to control in many cropping systems due to its prolific seed production (>42000 seeds plant^-1^) as well as its resistance to several common herbicides such as ACCase inhibitors, ALS inhibitors, EPSP synthase inhibitors, triazines, ureas and amides [2–4]. *C*. *virgata* is a key weed in sorghum production in the northern cropping region of Australia and in the southern and western regions of the country. The presence of this weed has also been reported in vineyards and orchards within these regions [5–7]. *C*. *virgata* is resistant to the EPSP synthase inhibitor herbicide and can produce up to 6000 seeds per plant [4–6].

In Australia, >80% of agricultural land is under conservation agriculture and no-tillage systems and accounts for more than 52 million ha [1]. In such systems, crop residue is retained on the soil surface whereby farmers gain many advantages, such as reduced soil erosion, reduced soil evaporation, increased microorganism activity and reduced weed seed germination [8–10]. In conservation agriculture, the application of pre-emergence (PRE) herbicides is highly recommended for the reduction of labor costs, the reduced need for costly post-emergence herbicides and an overall increase in weed suppression and control duration [11–13].

PRE herbicides play an integral role in weed control within conservation cropping systems. PRE herbicides such as imazethapyr [(Spinnaker), acetolactate synthase inhibitors], isoxaflutole [(Balance), 4-hydroxyphenyl-pyruvate dioxygenase inhibitors], S-metolachlor [(Dual Gold), cell division/ very long-chain fatty acid inhibitors], pendimethalin [(Rifle), microtubule assembly inhibitors] and prosulfocarb + metolachlor [(Boxer Gold), inhibitors of lipid synthesis and cell division/ very long-chain fatty acid inhibitors] are recommended for the control of grass weeds such as *E*. *colona* and *C*. *virgata* in many summer crops [14–19]. In this study, herbicides with different water solubility (mg/L at 20°C) [pendimethalin (0.33), isoxaflutole (6), prosulfocarb + metolachlor (16), metolachlor (480), and while metolachlor (200), isoxaflutole (145) and imazethapyr (1.4–173) tend to highly move with soil water [20]. Several reports suggest that a significant amount of PRE herbicide may be adsorbed by crop residue, whereby herbicide efficacy is reduced, according to its physicochemical properties [21–22]. For example, Banks and Robinson [23] found that 50% of metolachlor was adsorbed by wheat (*Triticum aestivum* L.) straw applied at 1 t ha^-1^. Khalil et al [22] observed that trifluralin tightly bound to wheat straw, and a small amount of this herbicide washed off from crop residue after rainfall (20 mm), while pyroxasulfone had easily lost bond with crop residue. Chauhan and Abugho [12] reported that some weeds such as *Cyperus iria* L. escape from PRE herbicide applications in the presence of rice residue. There remains a significant gap of information for the northern regions of Australia on the interaction of crop residue and PRE herbicides. Information on sorghum residue retention and PRE herbicide application can be used to develop precise and integrated weed management strategies.

The aim of this study was to evaluate the interaction effect of sorghum residue and PRE herbicides on emergence and biomass of *E*. *colona* and *C*. *virgata*.

## Materials and methods

### Seed description and soil preparation

In 2017, seeds of *E*. *colona* and *C*. *virgata* were collected from Gatton (27.43°S, 152.24°E), Queensland, Australia. For each weed species, 100 mature seeds were placed on the soil surface of plastic pots (10 cm diameter × 10 cm height) filled with field soil. The soil collected was sandy loam soil (2% organic matter, pH 7) from the research farm at the University of Queensland, Gatton. This soil was sieved and placed in an oven set at 100°C for a week to eliminate any background seed bank.

### Herbicide application and sorghum residue

**Fig 1:**
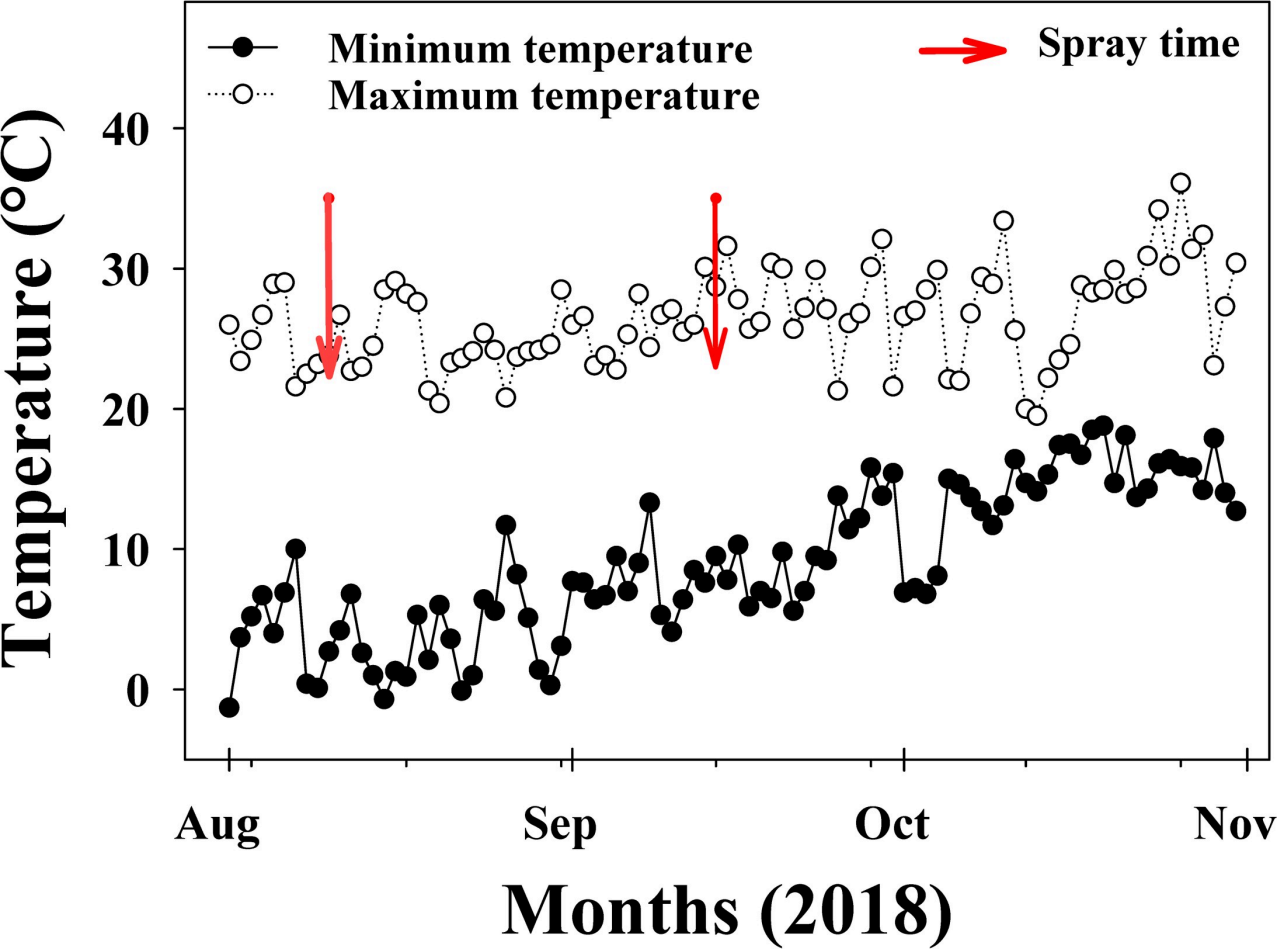
Minimum and maximum temperatures during the studies on the interaction of sorghum residue and pre-emergence herbicides on emergence and biomass of *Echinochloa colona* and *Chloris virgata* in a shade house at the Gatton Campus of the University of Queensland, Australia.

This study was conducted in 2018 in a shade house facility of the Queensland Alliance for Agriculture and Food Innovation (QAAFI), the University of Queensland, Gatton, Australia. The oven-dried (70°C for 72 h) small pieces (2 cm) of sorghum (cv. MR Bazley) residue (leaves and stems) at the field equivalent rates of 0, 3 and 6 t ha^-1^ were used to evaluate the interaction of varying rates of PRE herbicides and sorghum residue. PRE herbicides [imazethapyr (100 and 150 g a. i. ha^-1^), isoxaflutole (100 and 200 g a. i. ha^-1^), metolachlor (1.5 and 2.25 kg a. i. ha^-1^), pendimethalin (2.25 and 3.38 kg a. i. ha^-1^) and prosulfocarb + metolachlor (2.5 and 3.75 kg a. i. ha^-1^)] were spayed using a Research Track Sprayer. Flat fan nozzles (110015) were used and the total spray volume was 108 L ha^-1^. For each residue amount, there was a control (no-herbicide treatment). Each pot was daily sub-irrigated separately after herbicide treatment, and the soil was kept at field capacity. Minimum and maximum temperatures of the shade house during the study are given in Fig 1.

### Data collection

The study was terminated after 5 weeks of herbicide application. Emerged weed seedlings were counted at harvest. Weed biomass was collected by cutting the emerged seedlings at the soil surface before being placed in an oven set at 70°C for 72 h for drying [12].

### Statistical analyses

A factorial experiment (herbicide × sorghum residue) based on a randomized complete block design with three replications was used. The study was repeated once. The data for seedling emergence was pooled as no significant differences were observed between the two experimental runs. However, biomass data is presented separately for each experimental run due to significant differences between each experimental run. Before analysis (ANOVA), the data was subjected to the Shapiro-Wilk test and no transformation was needed. The Fisher’s protected Least Significant Differences (LSD) was used at probability 0.05 to evaluate the difference among means.

## Results

### Effect of PRE herbicides on emergence of *Echinochloa colona*

The interaction effect of herbicide treatment and sorghum residue was significant for the emergence of *E*. *colona* (Table 1). In the no-herbicide control, covering the seeds with 3 t ha^-1^ sorghum residue resulted in a 20% increase in the emergence of *E*. *colona* d in comparison with the no-residue treatment (41% emergence) with no significant differences observed between 3 and 6 t ha^-1^ sorghum residue treatments. Without crop residue, seedling emergence of *E*. *colona* was completely inhibited by isoxaflutole (both rates), metolachlor (both rates), pendimethalin (3.38 kg ha^-1^) and prosulfocarb + metolachlor (3.75 kg ha^-1^).

**Table 1:** Effect of residue amount and herbicide treatments on seedling emergence of *Echinochloa colona*.

Herbicide treatments	Residue amount (t ha^-1^)		
	0	3	6
	Seedling emergence (%)		
Control	41.3	51.7	51.3
Imazethapyr 100 g ha^-1^	12.3	21.0	34.3
Imazethapyr 150 g ha^-1^	5.3	12.3	27.3
Isoxaflutole 100 g ha^-1^	0.0	20.3	32.7
Isoxaflutole 200 g ha^-1^	0.0	0.0	0.0
Metolachlor 1.5 kg ha^-1^	0.0	32.0	29.7
Metolachlor 2.25 kg ha^-1^	0.0	27.6	27.0
Pendimethalin 2.25 kg ha^-1^	3.0	31.3	37.7
Pendimethalin 3.38 kg ha^-1^	0.0	23.3	32.0
Prosulfocarb + metolachlor 2.5 kg ha^-1^	2.0	46.7	44.0
Prosulfocarb + metolachlor 3.75 kg ha^-1^	0.0	33.0	41.3
LSD (0.05)	7.63		

In the no-residue cover treatment, seedling emergence was reduced by 70% following the application of imazethapyr at 100 g ha^-1^ in comparison with the no-herbicide treatment. Increasing sorghum residue from 3 to 6 t ha^-1^ resulted in 39% and 55% increases in seedling emergence at 100 and 150 g ha^-1^ of imazethapyr, respectively. In the 6 t ha^-1^ sorghum residue treatment, increasing the imazethapyr rate from 100 to 150 g ha^-1^ did not affect seedling emergence of *E*. *colona*.

Application of isoxaflutole at 200 g ha^-1^ resulted in complete inhibition of seedling emergence. In the isoxaflutole 100 g ha^-1^ treatment, increasing sorghum residue from 3 to 6 t ha^-1^ resulted in a 38% increment in the emergence of *E*. *colona*.

In the 3 t ha^-1^ sorghum residue treatment, application of metolachlor at 1.5 kg ha^-1^ reduced the emergence of this weed by 38% in comparison with the no-herbicide treatment. At 3 t ha^-1^ sorghum residue, increasing the metolachlor rate and sorghum residue did not affect the seedling emergence of *E*. *colona*.

At 3 t ha^-1^ sorghum residue, the application of 2.25 and 3.38 kg ha^-1^ pendimethalin resulted in 39% and 55% reductions in seedling emergence of *E*. *colona* in comparison with the no-herbicide treatment. Increasing the sorghum residue amount from 3 to 6 t ha^-1^ in the pendimethalin 3.38 kg ha^-1^ treatment resulted in a 27% increase in seedling emergence; however, this increment was not observed at the lower herbicide rate (i. e., 2.5 kg ha^-1^).

In the 3 and 6 t ha^-1^ sorghum residue treatments, spraying prosulfocarb + metolachlor at 2.5 kg ha^-1^ did not affect the emergence of this weed in comparison with the no-herbicide treatment. However, the higher rate of this herbicide treatment (i. e., 3.75 kg ha^-1^) reduced the seedling emergence of this weed by 36% and 19% at 3 and 6 t ha^-1^ sorghum residue amounts, respectively, in comparison to the no-herbicide treatment.

### Effect of PRE herbicides on emergence of *Chloris virgata*

The interaction between herbicide treatment and sorghum residue was significant on the emergence of *C*. *virgata* (Table 2). In the no-herbicide treatment (control), covering the seeds with 3 t ha^-1^ sorghum residue resulted in a 16% increase in the emergence of this weed in comparison with the no-residue treatment. Comparatively in the 6 t ha^-1^ sorghum residue treatment, seedling emergence of this weed reduced by 48% in comparison with the no-residue treatment. Without sorghum residue, seedling emergence of this weed was completely inhibited by the application of isoxaflutole, metolachlor, pendimethalin and prosulfocarb + metolachlor.

**Table 2:** Effect of residue amount and herbicide treatments on seedling emergence of *Chloris virgata*.

Herbicide treatments	Residue amount (t ha^-1^)		
	0	3	6
	Seedling emergence (%)		
Control	32.0	38.0	16.6
Imazethapyr 100 g ha^-1^	10.3	23.3	16.7
Imazethapyr 150 g ha^-1^	3.7	19.3	10.7
Isoxaflutole 100 g ha^-1^	0.0	1.0	3.3
Isoxaflutole 200 g ha^-1^	0.0	0.0	0.0
Metolachlor 1.5 kg ha^-1^	0.0	3.0	1.3
Metolachlor 2.25 kg ha^-1^	0.0	1.7	1.3
Pendimethalin 2.25 kg ha^-1^	0.0	3.0	4.3
Pendimethalin 3.38 kg ha^-1^	0.0	0.0	2.3
Prosulfocarb + metolachlor 2.5 kg ha^-1^	0.0	5.0	11.0
Prosulfocarb + metolachlor 3.75 kg ha^-1^	0.0	0.0	4.3
LSD (0.05)	3.29		

Compared with no-herbicide treatment, the seedling emergence of *C*. *virgata* reduced by 68% and 39% when imazethapyr 100 g ha^-1^ was applied with no-residue and 3 t ha^-1^ sorghum residue respectively. Increasing the sorghum residue amount from 3 to 6 t ha^-1^ reduced seedling emergence of this weed by 28% and 45% at 100 and 200 g ha^-1^ of imazethapyr, respectively.

In the 3 t ha^-1^ sorghum residue treatment, application of isoxaflutole (100 g ha^-1^), metolachlor (1.50 kg ha^-1^) and pendimethalin (2.25 kg ha^-1^) reduced seedling emergence of *C*. *virgata* by 97%, 92% and 92%, respectively, in comparison with the no-herbicide treatment.

At 3.75 kg ha^-1^ of prosulfocarb + metolachlor, the seedling emergence of *C*. *virgata* reduced by 74% at 6 t ha^-1^ sorghum residue in comparison to the no-herbicide treatment and no plant survived at 3 t ha^-1^ sorghum residue.

### Effect of PRE herbicides on biomass of *Echinochloa colona*

The interaction effect of herbicide treatment and sorghum residue was significant on the biomass of *E*. *colona* (Table 3). Although significant differences were observed between experimental runs, *E*. *colona* responded similarly to herbicide and sorghum residue treatments. In both experimental runs, addition of sorghum residue resulted in an increase in biomass in the no-herbicide treatment. In the no-residue treatment, imazethapyr 200 g ha^-1^ resulted in 82% and 61% reductions in biomass in the first and second experimental runs, respectively, compared with the no-herbicide treatment. In comparison with the no-residue treatment, retaining residue at 3 t ha^-1^ resulted in reductions in biomass by 39%, 36%, 30% and 44% at 100 g ha^-1^ of imazethapyr, 100 g ha^-1^ of isoxaflutole, 1.5 kg ha^-1^ of metolachlor and 2.25 kg ha^-1^ of pendimethalin, respectively, in the first experimental run. In the second experimental run, the corresponding values were 50%, 48%, 48% and 55%, respectively. In the abovementioned treatments (both experimental runs), increasing sorghum residue amount did not affect the biomass of this weed.

**Table 3:** Effect of residue amount and herbicide treatments on seedling biomass of *Echinochloa colona*.

Herbicide treatments	Residue amount (t ha^-1^)		
	0	3	6
	Biomass (g per pot)		
Experimental run I			
Control	0.39	0.61	0.54
Imazethapyr 100 g ha^-1^	0.22	0.37	0.39
Imazethapyr 150 g ha^-1^	0.07	0.27	0.36
Isoxaflutole 100 g ha^-1^	0.0	0.39	0.35
Isoxaflutole 200 g ha^-1^	0.0	0.0	0.0
Metolachlor 1.5 kg ha^-1^	0.0	0.43	0.37
Metolachlor 2.25 kg ha^-1^	0.0	0.38	0.36
Pendimethalin 2.25 kg ha^-1^	0.09	0.34	0.31
Pendimethalin 3.38 kg ha^-1^	0.0	0.33	0.35
Prosulfocarb + metolachlor 2.5 kg ha^-1^	0.17	0.49	0.50
Prosulfocarb + metolachlor 3.75 kg ha^-1^	0.0	0.46	0.47
LSD (0.05)	0.14		
Experimental run II			
Control	0.83	1.11	0.97
Imazethapyr 100 g ha^-1^	0.45	0.55	0.60
Imazethapyr 150 g ha^-1^	0.32	0.51	0.66
Isoxaflutole 100 g ha^-1^	0.00	0.57	0.43
Isoxaflutole 200 g ha^-1^	0.00	0.00	0.00
Metolachlor 1.5 kg ha^-1^	0.00	0.57	0.49
Metolachlor 2.25 kg ha^-1^	0.00	0.49	0.51
Pendimethalin 2.25 kg ha^-1^	0.35	0.50	0.58
Pendimethalin 3.38 kg ha^-1^	0.00	0.55	0.58
Prosulfocarb + metolachlor 2.5 kg ha^-1^	0.16	0.99	0.91
Prosulfocarb + metolachlor 3.75 kg ha^-1^	0.00	0.70	0.86
LSD (0.05)	0.12		

In the no-residue treatment, the biomass of *E*. *colona* was reduced by 77% and 56% at 2.25 kg ha^-1^ of pendimethalin and 2.5 kg ha^-1^ of prosulfocarb + metolachlor, respectively, compared with the no herbicide treatment in the first experimental run. Comparatively in the second experimental run, the biomass of this weed reduced by 58% and 81%, respectively. In the 6 t ha^-1^ residue treatment, application of prosulfocarb + metolachlor 3.75 kg ha^-1^ did not affect biomass of *E*. *colona* compared with the no-herbicide treatment.

### Effect of PRE herbicides on biomass of *Chloris virgata*

The interaction effect of herbicide treatment and sorghum residue was significant on the biomass of *C*. *virgata* (Table 4). Although significant differences were observed between experimental runs, this weed responded similarly to herbicide and sorghum residue treatments. In both experimental runs, addition of sorghum residue did not affect the biomass of this weed in the on-herbicide treatment.

**Table 4:** Effect of residue amount and herbicide on seedling biomass of *Chloris virgate*.

Herbicide treatments	Residue amount (t h-1)		
	0	3	6
	Biomass (g per pot)		
Experimental run I			
Control	0.35	0.56	0.48
Imazethapyr 100 g ha^-1^	0.12	0.44	0.42
Imazethapyr 150 g ha^-1^	0.11	0.37	0.33
Isoxaflutole 100 g ha^-1^	0.00	0.11	0.16
Isoxaflutole 200 g ha^-1^	0.00	0.00	0.0
Metolachlor 1.5 kg ha^-1^	0.00	0.20	0.12
Metolachlor 2.25 kg ha^-1^	0.00	0.12	0.12
Pendimethalin 2.25 kg ha^-1^	0.00	0.26	0.27
Pendimethalin 3.38 kg ha^-1^	0.00	0.00	0.27
Prosulfocarb + metolachlor 2.5 kg ha^-1^	0.00	0.36	0.37
Prosulfocarb + metolachlor 3.75 kg ha^-1^	0.00	0.24	0.29
LSD (0.05)	0.21		
Experimental run II			
Control	0.50	0.56	0.58
Imazethapyr 100 g ha^-1^	0.31	0.50	0.57
Imazethapyr 150 g ha^-1^	0.24	0.54	0.48
Isoxaflutole 100 g ha^-1^	0.00	0.23	0.34
Isoxaflutole 200 g ha^-1^	0.00	0.00	0.00
Metolachlor 1.5 kg ha^-1^	0.00	0.37	0.26
Metolachlor 2.25 kg ha^-1^	0.00	0.24	0.20
Pendimethalin 2.25 kg ha^-1^	0.00	0.42	0.50
Pendimethalin 3.38 kg ha^-1^	0.00	0.00	0.45
Prosulfocarb + metolachlor 2.5 kg ha^-1^	0.00	0.52	0.57
Prosulfocarb + metolachlor 3.75 kg ha^-1^	0.00	0.41	0.51
LSD (0.05)	0.13		

Application of different rates of imazethapyr (100 and 150 g ha^-1^) did not affect the biomass of this weed at 3 and 6 t ha^-1^ sorghum residue treatments in comparison with the no-herbicide treatment in both experimental runs. In the no-residue treatment, imazethapyr 100 g ha^-1^ reduced the biomass of this weed by 66% and 38% in the first and second experimental runs, respectively, in comparison with the no-herbicide treatment.

In the first experimental run at 3 t ha^-1^ sorghum residue, the biomass of *C*. *virgata* reduced by 80% and 64% following the application of 100 g ha^-1^ isoxaflutole and 1.5 kg ha^-1^ metolachlor, respectively, compared with the no herbicide treatment. Similarly, in the second experimental run, the biomass of this weed reduced by 59% and 34% respectively. In both runs, increasing the metolachlor rate did not affect the biomass of *C*. *virgata*.

In the 3 t ha^-1^ sorghum residue treatment, biomass of *C*. *virgata* reduced by 54% and 25% at 2.25 kg ha^-1^ of pendimethalin in the first and second experimental run, respectively, compared with the no-herbicide treatment. In the 6 t ha^-1^ sorghum residue treatment, application of pendimethalin at 2.25 and 3.38 kg ha^-1^ did not affect the biomass of *C*. *virgata* compared with the no-herbicide treatment.

In both experimental runs, retaining sorghum residue at 3 and 6 t ha^-1^ did not affect the biomass of *C*. *virgata* in the prosulfocarb + metolachlor 2.25 kg ha^-1^ treatment. In the 3 t ha^-1^ sorghum residue treatment, application of prosulfocarb + metolachlor at 3.75 kg ha^-1^ resulted in weed biomass reduction by 57% and 27% in the first and second experimental run, respectively, in comparison with the no-herbicide treatment.

## Discussion

The farming practice of crop residue use in covering soil surfaces to reduce evaporation rate and consequently increase soil moisture has been well established [8, 24]. Furthermore, it has been shown to improve moisture conductivity and better seed-soil contact, resulting in higher plant seedling emergence rate and growth [24–25]. In the current study, the seedling emergence of *E*. *colona* (16%) and *C*. *virgata* (20%) under no herbicide treatment slightly increased with the addition of sorghum residue at 3 t ha^-1^, in comparison with the no-residue treatment. Mutti et al. [26] stated that the emergence of *E*. *colona* reduced by 47% as a result of covering the seed with an amount of 8 t ha^-1^ sorghum residue. In the present study, increasing the sorghum residue amount from 3 to 6 t ha^-1^ in the absence of herbicides did not affect the seedling emergence of *E*. *colona* but the seedling emergence of *C*. *virgata* was reduced by 48%. In the no-herbicide treatment, sorghum residue retention (3 and 6 t ha^-1^) increased the biomass of *E*. *colona*. This was in contrast to the biomass of *C*. *virgata* which was not affected by sorghum residue retention. Germination ecology studies revealed that although both weeds have many similar requirements for germination, *E*. *colona* could germinate in a broad range of soil moisture and the emergence of *E*. *colona* and *C*. *vigata* completely inhibited at -1 and -0.6 MPa, respectively [4,6]. It could be concluded that the effect of crop residue on seedling emergence may vary according to species, dependent upon plant requirements for moisture, temperature, light and seed reserve resources for seedling emergence [3, 21, 27]. It has been reported than the retention of crop residue in conservation agriculture protects weed seeds from predators and physical decomposition [21, 28]. Furthermore, the increased soil water content and organic matter available within conservation agriculture systems may result in higher weed seed microbial decomposition [21, 29–31].

The effect of temperature on plant biomass production has been reported in several studies [32–34]. Although biomass was higher for both weeds in the second experimental run, both weeds responded similarly to herbicide and sorghum residue treatments in each experimental run. The increased biomass in the second experimental run could be attributed to the increase in temperature shown in Fig 1. Furthermore, microbial herbicide decomposition may increase with an increase in temperature, thereby affecting herbicide efficacy [35–36].

Several PRE herbicides have been registered for the control of many grass weeds in Australia. In the current study, without sorghum residue, the seedling emergence of both weeds was inhibited by 93%-100% with the application of isoxaflutole, metolachlor, pendimethalin and prosulfocarb + metolachlor. Similar results were obtained by Chauhan and Abugho [12], that in no-crop residue treatment, the emergence of *E*. *colona* was completely inhibited by applications of oxidiazon and pendimethalin.

In the no-residue treatment, imazethapyr had a lower effect on seedling emergence of both weeds in comparison with other herbicide treatments. Imazethapyr belongs to the imidazolinones family and is considered a low rate herbicide that can be absorbed by roots and shoots. Imazethapyr is highly water soluble with a low vapor pressure (<0.013 MPa) that presents a high risk of leaching. Furthermore, it has been reported that it is sensitive to photodegradation in water [37–38]. In the no-residue treatment, the lower efficacy of imazethapyr in comparison with other herbicides could be attributed to leaching as a sub-irrigation system was used in this study and photodegradation might have been enhanced in the presence of water.

Chauhan and Abugho [12] reported that retaining rice residue on the soil surface may reduce PRE herbicide efficacy on some species and cause evasion of chemical control in conservation agriculture systems. All herbicide treatments in our study showed a lower efficacy on *E*. *colona* and *C*. *virgata* in the presence of sorghum residue and both weeds produced more biomass when sorghum residue was retained. In conservation agriculture systems where weed seeds remain on the soil surface, retention of crop residue may act as a physical barrier to the amount of herbicide received on the soil surface. Khalil et al [39] reported a higher prosulfocarb, pyroxasulfone, and trifluralin interception when increasing the crop (wheat, barley, canola, chickpea and lupin) residue from 2 to 4 t ha^-1^ and lower herbicide efficacy with crop residue present. Furthermore, PRE herbicide efficacy may depend on herbicide physicochemical properties in conservation agriculture systems [13, 21, 22, 25]. For example, herbicides such as pendimethalin may be exposed to high volatilization and photodegradation with crop residue retention [40]. In systems with high soil organic matter, low soluble herbicides such as pendimethalin may have higher dissipation rates due to strong soil binding and exposure to higher microbial degradation [41–42]. It may be concluded that despite the many benefits of crop residue in conservation agriculture systems, the amount of crop residue on the soil surface should be adjusted for adequate weed management strategies. The use of surfactants or the changing of herbicide formulation for low interception rates could improve PRE herbicide efficacy in conservation agriculture systems [21, 43].

Lower PRE herbicide efficacy in the presence of crop residue may encourage a higher herbicide application rate from users. In the current study, increasing the isoxaflutole rate from 100 to 200 g ha^-1^ resulted in complete inhibition of emergence of both weeds. The risk of the evolution of weed resistance to herbicides due to increased application rates has been well documented [44–46] and should not be neglected. Furthermore, the higher usage of herbicides and absorption of herbicides by crop residue may also result in the occurrence of phytotoxicity in the next crop [47].

## Conclusions

Australian farmers gain many advantages from retaining crop residue on the soil surface. In conservation agriculture systems, PRE herbicides are a major weed management practice. The results of this study showed that the efficacy of PRE herbicides may be reduced by crop residue retention. Herbicides in the presence of crop residue may result in high interception rates, leaching, photodegradation and microbial dissipation based on their physicochemical properties. In such systems, the amount of crop residue on the soil surface should be adjusted according to the nature of the PRE herbicides to avoid a high load and to achieve adequate weed control. In future studies, the feasibility of the application of more efficient herbicides with new formulation and a lower interception rate in the presence of crop residue based on weather conditions, such as temperature and precipitation should be evaluated.
