# Correction: Effects of sorghum residue in presence of pre-emergence herbicides on emergence and biomass of *Echinochloa colona* and *Chloris virgata*

**DOI:** 10.1371/journal.pone.0265522

**Published:** 2022-03-10

**Authors:** Ahmadreza Mobli, Abhimanyu Rinwa, Sahil, Bhagirath Singh Chauhan

The PRE herbicide names metolachlor and prosulfocarb + s-metolachlor appear incorrectly throughout the article. The correct names are s-metolachlor and prosulfocarb + s-metolachlor.

Additionally, the PRE herbicide doses appear incorrectly throughout the article. The correct doses are imazethapyr (70 and 105 g a. i. ha^-1^), isoxaflutole (75 and 150 g a. i. ha^-1^), s-metolachlor (1.44 and 2.16 kg a. i. ha^-1^), pendimethalin (0.99 and 1.49 kg a. i. ha^-1^) and prosulfocarb + s-metolachlor (2.3 and 3.45 kg a. i. ha^-1^).
